# Amino Acid Starvation Sensitizes Resistant Breast Cancer to Doxorubicin-Induced Cell Death

**DOI:** 10.3389/fcell.2020.565915

**Published:** 2020-10-15

**Authors:** Mark Thomas, Tanja Davis, Theo Nell, Balindiwe Sishi, Anna-Mart Engelbrecht

**Affiliations:** ^1^Department of Physiological Sciences, Faculty of Natural Sciences, Stellenbosch University, Stellenbosch, South Africa; ^2^African Cancer Institute (ACI), Department of Global Health, Faculty of Medicine and Health Sciences, Stellenbosch University, Tygerberg, South Africa

**Keywords:** nutrient starvation, amino acids, doxorubicin, breast cancer, sensitization

## Abstract

Many clinical trials are beginning to assess the effectiveness of compounds known to regulate autophagy in patients receiving anti-cancer chemotherapy. However, autophagy inhibition, through exogenous inhibitors, or activation, through starvation, has revealed conflicting roles in cancer management and chemotherapeutic outcome. This study aimed to assess the effect of amino acid starvation on doxorubicin-treated breast cancer cells by assessing the roles of autophagy and apoptosis. An *in vitro* breast cancer model consisting of the normal breast epithelial MCF12A and the metastatic breast cancer MDAMB231 cells was used. Apoptotic and autophagic parameters were assessed following doxorubicin treatments, alone or in combination with bafilomycin, ATG5 siRNA or amino acid starvation. Inhibition of autophagy, through ATG5 siRNA or bafilomycin treatment, increased caspase activity and intracellular doxorubicin concentrations in MCF12A and MDAMB231 cells during doxorubicin treatment. While amino acid starvation increased autophagic activity and decreased caspase activity and intracellular doxorubicin concentrations in MCF12A cells, no changes in autophagic parameters or caspase activity were observed in MDAMB231 cells. Our *in vivo* data showed that 24 h protein starvation during high dose doxorubicin treatment resulted in increased survival of tumor-bearing GFP-LC3 mice. Results from this study suggest that short term starvation during doxorubicin chemotherapy may be a realistic avenue for adjuvant therapy, especially with regards to the protection of non-cancerous cells. More research is however, needed to fully understand the regulation of autophagic flux during starvation.

## Introduction

Solid tumors make up the majority of all human cancers. Once solid neoplasms become established they can partially adapt to local micro-environmental shortages in nutrient supply by increasing autophagy (Mathew et al., 2007). Cellular nutritional status is closely associated with autophagy and several dietary factors are known to promote autophagy induction, one of the most effective being the restriction of dietary calorie intake. While dietary habits are also linked to cancer risk and progression (Popkin, 2007), caloric restriction exhibits a promising ability to extend the lifespan of patients (Heilbronn and Ravussin, 2005) and facilitate tumor suppression (Kritchevsky, 2003). The application of short term starvation protocols in patients receiving high doses of chemotherapy has proven successful in reducing side effects in these patients (Safdie et al., 2009). In a cell culture and neuroblastoma mouse xenograft model, normal cells placed on a similar starvation protocol were shown to benefit from differential protection compared to cancer cells during high dose chemotherapy regimens (Raffaghello et al., 2008). Mice starved for 48 h had reduced chemotoxicity following high dose treatment, whereas mice fed *ad libitum* were 50% more likely to die. Tumor cell death was not compromised by the starvation protocol. The underlying mechanisms responsible for this differential protection of non-cancer cells are not yet fully understood.

Autophagy has been reported to confer resistance onto apoptosis-deficient cancer cells under metabolic stress by delaying the onset of necrotic cell death (Degenhardt et al., 2006; Sutton et al., 2019). Similarly, autophagy has also been reported to protect Caco-2 cells following exposure to toxins released by *Vibrio cholera* by engulfing and sequestering the toxins in lysosomal compartments (Gutierrez et al., 2007). More recently, high mobility group box 1 (HMGB1) release following chemotherapy-induced damage to leukemia cells caused a protective autophagy response (Liu et al., 2011a), strengthening the possibility that damage-associated molecular pattern molecule (DAMP) release during chemotherapy can increase autophagy to grant a defensive reaction (Liu et al., 2011b). In this way, damage caused by cytotoxic agents could directly result in an increased autophagic response.

Based on the premise that autophagy can promote tumor survival, it is believed that targeted and specific inhibition of autophagy could be a promising therapeutic avenue. Several *in vitro* studies have illustrated the potential of class-III phosphatidylinositol-3-kinase inhibitors such as 3-methyladenine, which prevent the formation of autophagosomes, in cancer therapy (Kanzawa et al., 2004). However, while starvation of a cervical cancer cell line resulted in apoptosis in the presence of this inhibitor (Boya et al., 2005), 3-methyladinine prevented tamoxifen-induced apoptosis in breast cancer cells (Bursch et al., 1996). Agents such as bafilomycin A1 (Baf), hydroxychloroquine and monensin (all of which prevent lysosomal fusion with autophagosomes) triggered apoptosis in HeLa cells during nutrient depletion (Boya et al., 2005), whilst Baf was also able to impede the protective effect of autophagy in several cancer lines undergoing radiation therapy (Paglin et al., 2001).

Even though Doxorubicin (Dox) is possibly the most effective anti-cancer agent available to date, it is also cytotoxic and can lead to cardiotoxicity as a result of its cumulative and dose-dependent effects (Swain et al., 2003). More effective strategies are needed to increase efficacy and protect non-cancer cells from off-target cytotoxicity. It is now also known that many anti-cancer agents and therapies increase autophagy levels in treated cancer cells at certain doses (Wu et al., 2006; Park et al., 2008). Transient, rapid and unpredictable alterations in autophagic flux could modify the way tumors respond to chemotherapy and supposedly interfere with or even augment therapy outcomes in unexpected ways. This study therefore aimed to establish the relative sensitivity of MDAMB231 and MCF12A cells to Doxorubicin, followed by the assessment of autophagy, apoptosis and the cell cycle. Furthermore, the efficiency of amino acid starvation in combination with Dox treatment was also assessed. Finally, a tumor-bearing mouse model was employed to assess whether protein starvation can sensitize tumors to Dox therapy.

## Materials and Methods

### Cell Culture

Experiments were performed using two cell lines: the human metastatic mammary carcinoma cell line, MDAMB231, obtained from American Type Culture Collection (Rockville, MD, United States) and the human non-tumourigenic breast epithelial cell line, MCF12A, obtained from the University of Cape Town. The MCF7 cell line, used in selected experiments, was a donation from the University of the Western Cape.

During routine maintenance, cells were grown as monolayers in Glutamax-Dulbecco Modified Eagles Medium (DMEM; Celtic Molecular Diagnostics, Cape Town, South Africa) supplemented with 10% fetal bovine serum (FBS; Sigma Chemical Co., St Louis, MO, United States) at 37°C in a humidified atmosphere with 5% CO_2_. MCF12A cell’s growth medium was supplemented with Hams F12 medium (1:1), 0.5 μg/ml hydrocortisone, 10 μg/ml insulin and 20 ng/ml epithelial growth factor (EGF). All supplements were obtained from Sigma Chemical Co., St Louis, MO, United States.

Cells were first allowed to proliferate in T75 flasks (75 cm^2^ flasks, Greiner Bio One, Germany) until confluency of approximately 80%, where after cells were subcultured into appropriate treatment plates or dishes. All experiments were performed using exponentially growing cells.

### Drug Treatments

The following groups were used for the purpose of this study: (i) a control group, (ii) a Dox group, (iii) a siRNA control group, (iv) a siRNA control Dox group, (v) an ATG5 siRNA Dox group (ATG5 siRNA transfection 24 h prior to Dox treatment for 24 h), (vi) a Baf group and (vii) a Baf Dox group (Baf treatment 6 h prior to analysis, Dox treatment for 24 h).

Dox hydrochloride (D1515, Sigma Chemical Co., St Louis, MO, United States) stock solutions, as well as further dilutions to a concentration of 1 μM, was prepared in amino acid free medium. Bafilomycin (B1793, Sigma Chemical Co., St Louis, MO, United States) was dissolved in dimethyl sulfoxide (DMSO) to prepare stock solutions, while further dilutions to a concentration of 10 nM was performed in amino acid free medium. Based on previous experience (unpublished results), DMSO at this concentration (0.00625%) does not have any cytotoxic effects on MCF12A and MDAMB231 cells. For the siRNA group, please see the next section. For experiments conducted in the absence of amino acids, amino acid free culture medium (Highveld Biological Pty.) was used.

### ATG5 siRNA Transfection

Cells were transfected using a reverse transcription protocol into 60 mm petri dishes. ATG5 siRNA duplex (20 pmol, Cell Signaling, Danvers, MA, United States, lSilence^®^ Atg5 siRNA I #6345) was diluted into 250 μl transfection medium (containing no antibiotics or serum), where after 2 μl of Lipofectamine^TM^ RNAiMAX (13778075; Invitrogen^TM^, United States) was added. A volume of 250 μl from this suspension was added to each petri dish and allowed to incubate for 20 min. MCF12A cells (100 000) or MDAMB231 cells (80 000) were then plated into the culture dishes containing the RNAi duplex-Lipofectamine RNAiMAX complexes to have a final volume of 2 ml and gently mixed. Cells were incubated at 37°C until ready to treat, 48 h later. Stealth RNAi (STEALTH RNAI NEG CTL MED GC, 12935300; Invitrogen^TM^, United States) was used as a negative control.

### Caspase 3/7 Activity

Caspase-3/7 activity was measured using the Caspase-Glo^®^ 3/7 assay (Promega, Madison, WI, United States). MCF12A cells (15 000) or MDAMB231 cells (10 000) were plated 48 h prior to treatment in white-walled 96-well plates in 100 μl culture medium. In experiments where transfection was necessary, cells were reverse transfected during plating. Following treatment, 100 μl (1:1) of Caspase-Glo^®^ 3/7 working reagent was added to each well, and the plates mixed at 500 rpm for 30 s. The plates were then incubated at 22°C for 1 h in the dark and the luminescence measured in a luminometer (GloMax^TM^ 96 Microplate Luminometer, Promega).

### Trypan Blue Assay

Trypan blue cell viability was assessed using the Countess^TM^ Automated Cell Counter (Invitrogen, United States). MCF12A cells (100 000) or MDAMB231 cells (80 000) were plated into 60 mm culture dishes 48 h prior to treatment. Following treatment, cells were trypsinised and pelleted, where after a 10 μl cell suspension was mixed with 10 μl 0.4% trypan blue and 10 μl loaded into a Countess^TM^ chamber slide. Trypan blue positivity was calculated automatically and expressed as the total number of trypan blue positive cells, total number of trypan blue negative cells and absolute total cell number.

### Western Blotting

Following treatment, plates containing cell monolayers were rinsed three times in 5 ml pre-lysis buffer (20 mM Tris-HCl, pH 7.4, 137 mM NaCl, 1 mM CaCl_2_, 1 mM MgCl_2_ and 0.1 mM sodium orthovanadate). Total cell protein was extracted by incubating cells on ice for 10 min in 1 ml radioimmunoprecipitation (RIPA, pH 7.4) buffer containing: 2.5 mM Tris–HCl, 1 mM EDTA, 50 mM NaF, 50 mM NaPPi, 1 mM dithiothreitol, 0.1 mM phenylmethylsulfonyl fluoride (PMSF), 1 mM benzamidine, 4 mg/ml SBTI, 10 mg/ml leupeptin, 1% NP40, 0.1% SDS and 0.5% Na-deoxycholate. Adherent cells were harvested from culture dishes by scraping, where after whole cell lysates were sonicated and centrifuged (8,000 rpm for 10 min; 4°C). Protein content was quantified using the Bradford protein determination method (Bradford, 1976) directly before preparation of cell lysates.

Fourty microgram protein extracts were prepared in Laemmli sample buffer and boiled for 5 min (95°C) prior to their separation on 12% polyacrylamide gels for LC3 II and caspase 3 and 10% polyacrylamide gels for beclin 1 by sodium dodecyl sulphate polyacrylamide gel electrophoresis (SDS-PAGE). Gels were run for 60 min at 130 V (constant) and 400 mA (Mini Protean System, Bio-Rad, United States). Following SDS-PAGE, proteins were transferred to polyvinylidine fluoride (PVDF) membranes (Immobilon, Millipore, United States) using a semi-dry electrotransfer system (Bio-Rad, United States) for 60 min at 15 V and 0.5 A. To prevent non-specific binding, membranes were blocked in 5% (w/v) fat-free milk in 0.1% Tris Buffered Saline-Tween20 (TBS-T) for 2 h at room temperature with gentle agitation. Membranes were incubated with LC3 primary antibody, caspase 3 primary antibody or beclin 1 primary antibody (all 1:1000; Cell Signaling, Danvers, MA, United States) overnight at 4°C. Goat anti-rabbit (Amersham Biosciences, United Kingdom, and Dako Cytomation, Denmark) horseradish peroxidase (HRP)-conjugated secondary antibody was added and incubated for 1 h at room temperature with gentle agitation. Antibodies were detected with the LumiGLO Reserve^TM^ chemiluminescent substrate kit (KPL, Inc., United States) and exposed to autoradiography film (Hyperfilm, Amersham Biosciences, United Kingdom). Exposed bands were visualized and quantified by densitometry using the UNSCAN-IT© densitometry software (Silk Scientific Corporation, Utah, United States). All bands were expressed as optical density readings relative to a control present on the same blot.

### Cell Cycle Analysis

Flow cytometric analysis of the cell cycle was performed using the CycleTEST^TM^ PLUS DNA Reagent kit (Becton Dickinson, San Jose, CA, United States). MCF12A cells (200 000) or MDAMB231 cells (150 000) were plated in T25 culture flasks 48 h prior to treatment. Prior to analysis, cells were trypsinised and the cell suspensions centrifuged at 400 × *g* for 5 min at room temperature. Cells were washed in phosphate buffered saline (PBS) and 250 μl trypsin buffer was added to each tube and allowed to react for 10 min at room temperature. Thereafter, 200 μl of trypsin inhibitor and RNase buffer was added for 10 min, followed by 200 μl ice cold propidium iodide stain solution on ice in the dark for a further 10 min. Samples were filtered through a 50 μm nylon mesh. Sample fluorescence was acquired using flow cytometry within 30 min and results obtained with ModFit LT software (Verity software house, Inc., ME, United States.) on a BD FACSAria I. At least 30 000 list-mode data events were acquired for each sample. A 585/42 bandpass filter was used to analyze light emitted between 564 and 606 nm by stained cells. ModFit LT software was used to determine the percentage of cells in the g0/g1, s and g2/m phases. Mean percentages from three independent experiments were used to perform statistical comparisons.

### Hoechst Nuclear Staining (Analysis of Pyknosis and Karyorrhexis)

To differentiate between normal nuclear morphology and apoptosis, characterized as nuclear condensation and fragmentation, staining with the DNA dye Hoechst 33342 (Sigma Chemical Co., St Louis, MO, United States) was employed. MDAMB2321, MCF12A and MCF7 cells were cultured and maintained as described previously, where after 60 000 MCF12A cells, 45 000 MDAMB231 cells and 45 000 MCF7 cells were plated into 35 mm culture dishes containing coverslips 48 h prior to treatment. Thereafter, the coverslips were removed, placed over glass slides, and washed with 100 μl ice cold PBS. The coverslips were treated with 500 μl ice cold acetone:methanol (1:1) and incubated at 4°C for 10 min. The fixative was removed and coverslips washed with PBS. A volume of 100 μl Hoechst in a 1:200 dilution (50 μg/ml) in sterile PBS was added directly onto the coverslips and incubated in the dark for 10 min. Coverslips were washed with PBS at room temperature before being mounted. Cells were viewed immediately using a Nikon E-400 fluorescence microscope (Nikon Microscopes, Kobe, Japan) and images acquired using a Nikon DMX1200 color digital camera (Nikon Microscopes, Kobe, Japan) with ACT-I software. Three independent experiments were conducted and four representative regions of each condition were acquired per experiment. At least 200 cells were analyzed per region. For each image, the number of condensed/fragmented nuclei was counted and expressed as a percentage of the total number of nuclei counted. In this manner, the percentage apoptosis was determined for each experimental condition.

### Dox and Lysosomal-Associated Membrane 2A (LAMP-2A) Fluorescence Imaging

MCF12A cells (12 000) and MDAMB231 cells (10 000) were seeded into 8-well Nunc^TM^ chambered plates (Nalge Nunc, Rochester, NY, United States). Cell monolayers were washed three times with sterile PBS before being fixed and permeabilised with an ice cold 1:1 methanol:acetone mixture for 10 min at 4°C. After being left to air dry for 20 min in the dark, cells were washed three times in sterile PBS and non-specific binding blocked by incubation with 5% donkey serum for 20 min. Cells were then incubated with anti-LAMP-2A primary antibody (Cell Signaling, MA, United States) for 90 min. Thereafter, a FITC-bound secondary antibody was added for 30 min and nuclei counter-stained using Hoechst 33342 (10 mg/ml in a 1:200 dilution) for 10 min at 4°C. No additional steps were required for visualization of Dox since the compound exhibits strong autofluorescence. Images were acquired with an Olympus Cell^∧^R fluorescence 1X81 inverted microscope (Olympus Biosystems, Germany) using an F-view II camera for image acquisition and Cell^∧^R software for image processing.

### Lysotracker^TM^(Flow Cytometry)

MCF12A cells (200 000) or MDAMB231 or MCF7 cells (150 000) were plated into T25 culture flasks 48 h prior to treatment. Prior to analysis, cells were trypsinised and the cell suspensions centrifuged at 400 × *g* for 5 min at room temperature. Cells were then washed once in PBS. Lysotracker^TM^ (Invitrogen^TM^, United States) was prepared in PBS (1:10 000) immediately before use. All treated samples were split 1:1, where after cell suspensions were centrifuged at 400 × *g* for 5 min at room temperature. For each sample, one cell suspension was stained while the other was left unstained. For staining, the pellets were resuspended in 250 μl fresh Lysotracker^TM^ /PBS and incubated for 10 min at room temperature before analysis using flow cytometry (BD FACSAria I). At least 10,000 cells were collected using a 488 nm laser and 610LP, 616/23BP emission filters. Values obtained for the unstained samples were then subtracted from those obtained for the stained samples.

### Tumor Establishment and Animal Protocols

The protocols in this study were carried out according to the guidelines for the care and use of laboratory animals implemented at Stellenbosch University (2009B02004). Eight week-old female C57BL6 mice (Stellenbosch University animal facility) or GFP-LC3 mice (kindly donated by Noboru Mizushima, Department of Cell Biology, National Institute for Basic Biology, Okazaki, Japan) were used in this study. The mice were maintained on standard chow diet and tap water before beginning the experiment. Mice were inoculated subcutaneously on the left pad of the fourth mammary gland with 200 μl of 2.5 × 10^5^ E0771 cells suspended in Hanks Balanced Salt Solution (Sigma Chemical Co., St Louis, MO, United States), using a 23-gauge needle. This protocol was adapted from Ewens et al. (2006). Small tumors were evident by days 12–14 and grew to reach approximately 230 mm^2^ in volume by day 33.

### Drug Preparation and Administration

Doxorubicin hydrochloride (D1515, Sigma Chemical Co., St Louis, MO, United States) was dissolved in Hanks Balanced Salt Solution (Sigma Chemical Co., St Louis, MO, United States). Volumes were prepared to reflect the exact concentration required per kilogram (kg) of body weight for each mouse on the day of injection. Doxorubicin treatment was initiated on day 33 and was administered twice over a period 3 days (i.e., on day 33 and day 35) by means of i.p. injections. Mice were restrained by the scruff method and 100 μl drug suspensions were injected into the right caudal thigh (avoiding the femur and sciatic nerve) of each mouse using a 23-gauge needle. Control mice were injected with the vehicle only. Immediately following doxorubicin treatment, tumor-bearing mice were placed on either a control diet containing protein (Research Diets, Inc., New Brunswick, NJ, United States) or a diet free of protein (Research Diets, Inc., New Brunswick, NJ, United States) for a total of 24 h. These two diets were isocaloric. For the 24 h period in between interventions, mice received the control diet containing protein. Tumor size was monitored every 2 to 3 days by making measurements in two perpendicular dimensions parallel with the surface of the mice using digital calipers. The body weight of the mice was monitored twice weekly. To assess intratumour caspase cleavage, FLIVO^TM^ *in vivo* apoptosis tracers (Immunochemistry Technologies LLC, MN, United States) were used. The SR FLIVO^TM^ red dye was prepared according to the manufacturer’s protocol and 100 μl was injected into the tail vein of mice after appropriate treatments were completed. After 1 h, whole tumors were excised, digested and analyzed using flow cytometry on the BD FACSAria I.

### Statistical Analysis

Data from at least three independent experiments were analyzed. Significant differences between time points and treatment groups were analyzed using either a one or two-way analysis of variance (ANOVA), together with a Bonferonni *post hoc* test. All statistical analyses were performed using Graphpad Prism version 5.01 (Graphpad Software, Inc, San Diego, CA, United States). All values are presented as means ± standard error of the mean (SEM), and the minimum level of significance was accepted as *p* < 0.05.

## Results

### MDAMB231 Cells Are More Resistant to Dox Treatment Compared to MCF12A Cells

**FIGURE 1:**
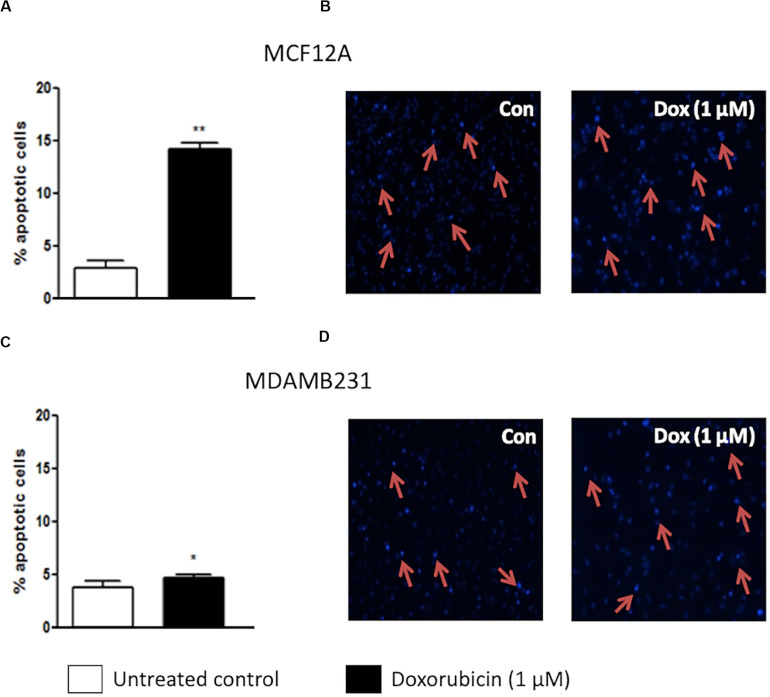
Percentage apoptotic cells in MCF12A and MDAMB231 cultures. **(A)** Bar graph indicating the percentage of nuclei that represents morphological changes characteristic of apoptosis in MCF12A cells, **(B)** representative image of MCF12A cells stained with Hoechst, **(C)** bar graphs indicating the percentage of nuclei that represents morphological changes characteristic of apoptosis in MDAMB231 cells, and **(D)** representative images of MDAMB231 cells stained with Hoechst. Red arrows demonstrate apoptotic nuclear features. Each value represents the mean ± SEM of at least three independent determinations. Con, control; *p < 0.05; **p < 0.01.

Dox treatment significantly increased apoptosis in MCF12A cells (Figure 1A). Approximately 15% of MCF12A cells presented with morphological changes to their nuclei (Figures 1A,B). MDAMB231 cells on the other hand displayed a relative resistance to apoptosis following treatment with Dox (Figure 1C). A very small, but statistically significant percentage of MDAMB231 cell’s nuclei presented with morphological changes characteristic of apoptosis (Figures 1C,D).

### Dox Treatment Increased LC3 II, but Not Beclin 1 Levels in MCF12A and MDAMB231 Cells

**FIGURE 2:**
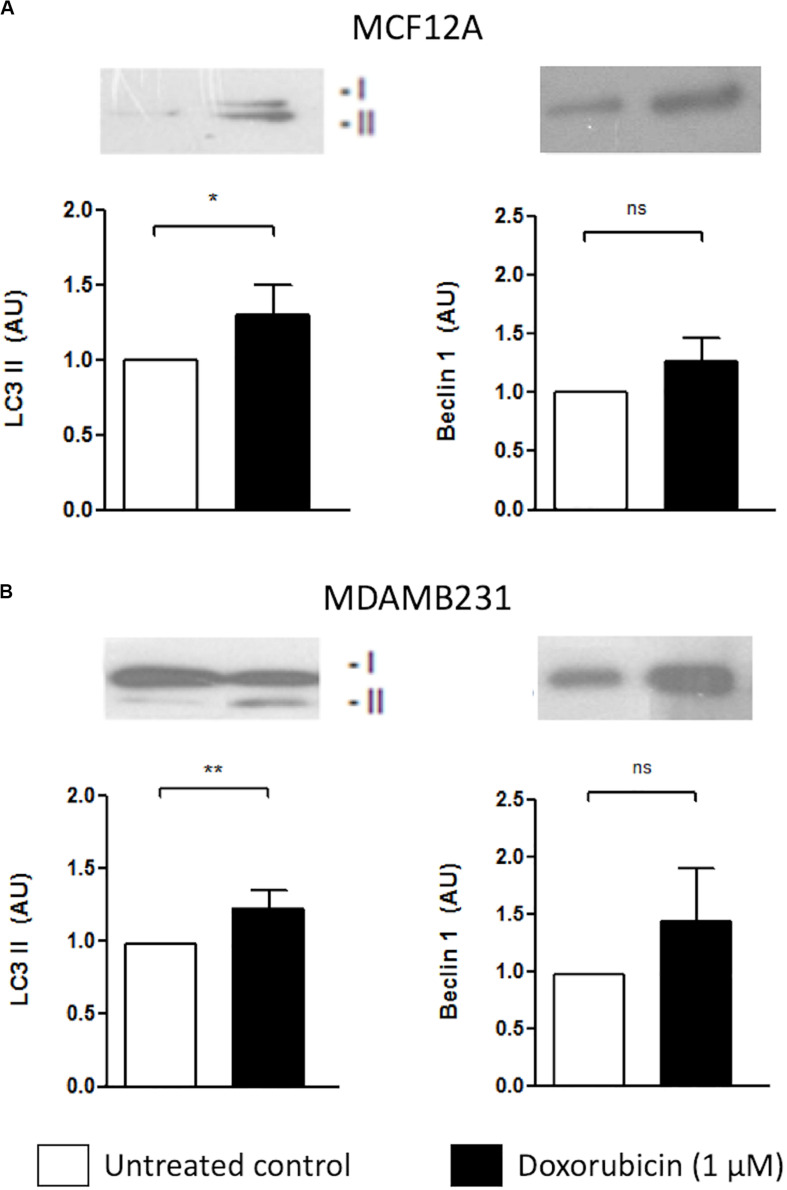
LC3 II and Beclin 1 protein levels in MCF12A and MDAMB231 cells treated with Dox. Representative western blots and bar graphs showing densitometric representation of LC3-II and Beclin-1 protein levels in MCF12A **(A)** and MDAMB231 **(B)** cells. Each value represents the mean ± SEM of at least three independent determinations. AU, arbitrary units; Con, control; *p < 0.05; **p < 0.01; ns, no significance.

Both MCF12A and MDAMB231 cells responded to the presence of Dox by increasing LC3 II protein levels (Figure 2). Although there was a slight increase in beclin-1 expression in both cell lines, these changes did not reach statistical significance.

### Caspase Activity Increased Following Treatment With Dox and Dox Combinations

**FIGURE 3:**
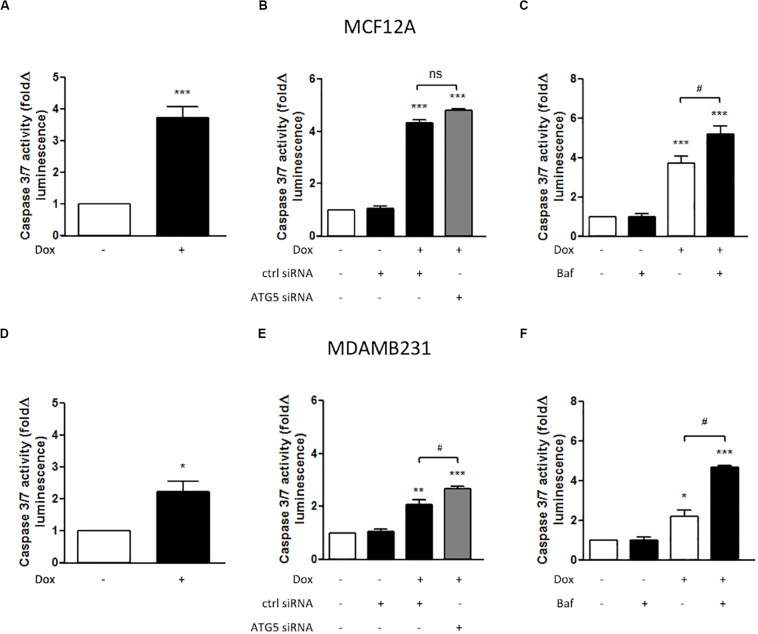
Caspase 3/7 activity in MCF12A and MDAMB231 cells following Dox treatment and autophagy inhibition. Caspase 3/7 activity in MCF12A cells following treatment with Dox alone **(A)**, in combination with ATG5 siRNA-mediated autophagy inhibition **(B)**, and in combination with Baf-mediated autophagy inhibition **(C)**. Caspase 3/7 activity in MDAMB231 cells following treatment with Dox alone **(D)**, in combination with ATG5 siRNA-mediated autophagy inhibition **(E)**, and in combination with Baf-mediated autophagy inhibition **(F)**. Results represent fold change in luminescence in cultures incubated with Dox vs. untreated cells. Images were obtained using a 10× objective with each value representing the mean ± SEM of at least three independent determinations. *p < 0.05; **p < 0.01; ***p < 0.001; ^#^*p* < 0.05, ns, no significance.

MCF12A cells displayed significantly increased caspase 3/7 activity following treatment with Dox for 24 h (Figure 3A). MDAMB231 cells also displayed a more pronounced (and significant) increase in caspase 3/7 activity following Dox treatment (Figure 3B). ATG5 siRNA transfection was used to inhibit autophagy. In MCF12A cells treated with Dox, ATG5 siRNA did not alter caspase 3/7 activity (Figure 3B), whereas in MDAMB231 cells, ATG5 siRNA significantly increased caspase 3/7 activity when administered in combination with Dox (Figure 3E). No changes in caspase activity was observed in either cell line when ATG5 siRNA was administered alone (Supplementary Figure 1). Treating either MCF12A or MDAMB231 cells with Baf (10 nM) 6 h prior to analysis greatly increased caspase 3/7 activity when these cells were also treated with Dox (Figures 3C,F).

### Baf Increased Detectable Levels of Intracellular Dox in MCF12A and MDAMB231 Cells Treated With Dox

**FIGURE 4:**
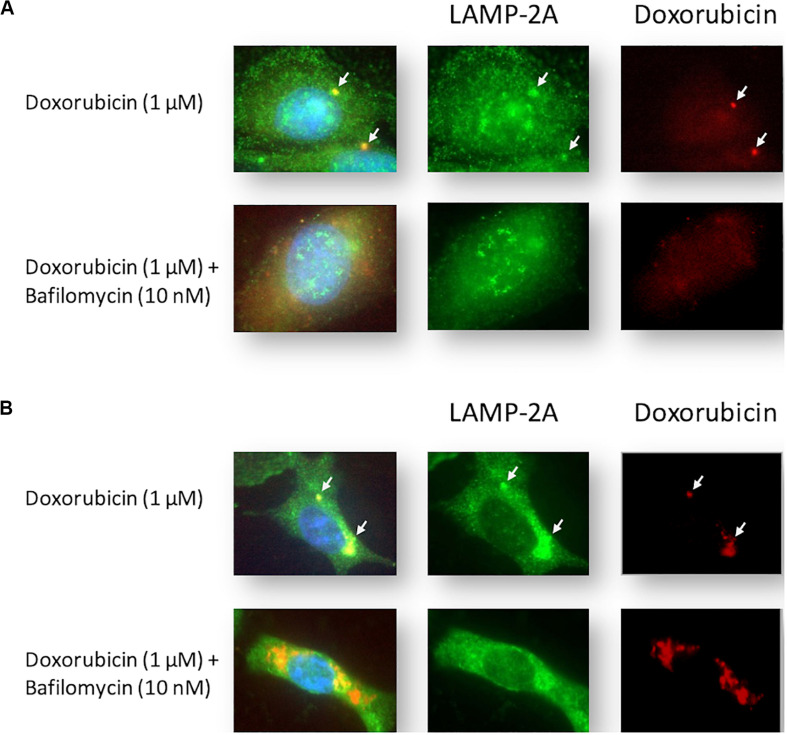
Intracellular localization of LAMP-2A and Dox in Dox-treated MCF12A and MDAMB231 cells following autophagy inhibition. Representative images of LAMP-2A staining (green) and Dox (red) within MCF12A **(A)** and MDAMB231 **(B)** cells in the presence of Dox alone or in combination with Baf. Arrows highlight regions of localized Dox accumulation overlapping with regions of punctate LAMP-2A signal. Comparative control groups (cells treated with Dox in the presence of amino acids) are shared between Figure 4 and Figure 7 since treatment groups from both figures were performed together in one experiment. Images were obtained using a 40× objective.

Cells were stained with LAMP-2A during Dox treatment. Intracellular localization of Dox was tracked by exploiting the compound’s autofluorescence, a technique that has been utilized successfully in MDAMB231 and other cell lines (Li et al., 2010). MCF12A cells treated with Dox displayed diffuse intracellular red fluorescence associated with Dox (Figure 4A). Notably, small localized regions of intense red fluorescence were observed in most Dox-treated MCF12A cells (arrows), but were completely absent in cells that had also been treated with Baf. MDAMB231 cells treated with Dox displayed intense localized regions of intracellular red fluorescence associated with Dox (Figure 4B). This red fluorescence was associated with pooled punctate LAMP-2A signal (arrows) in Dox-treated MDAMB231 cells. Notably, LAMP-2A fluorescence appeared more diffuse if Baf was added. Importantly, MDAMB231 cells treated with Dox and Baf had significantly more observable red fluorescence within the cytoplasmic regions of these cells. However, unlike MCF12A cells, there was no Dox associated with the nuclear regions of these cells.

### Depleting Culture Medium of Amino Acids During Dox Treatment Protects MCF12A, but Not MDAMB231 Cells From Apoptosis

**FIGURE 5:**
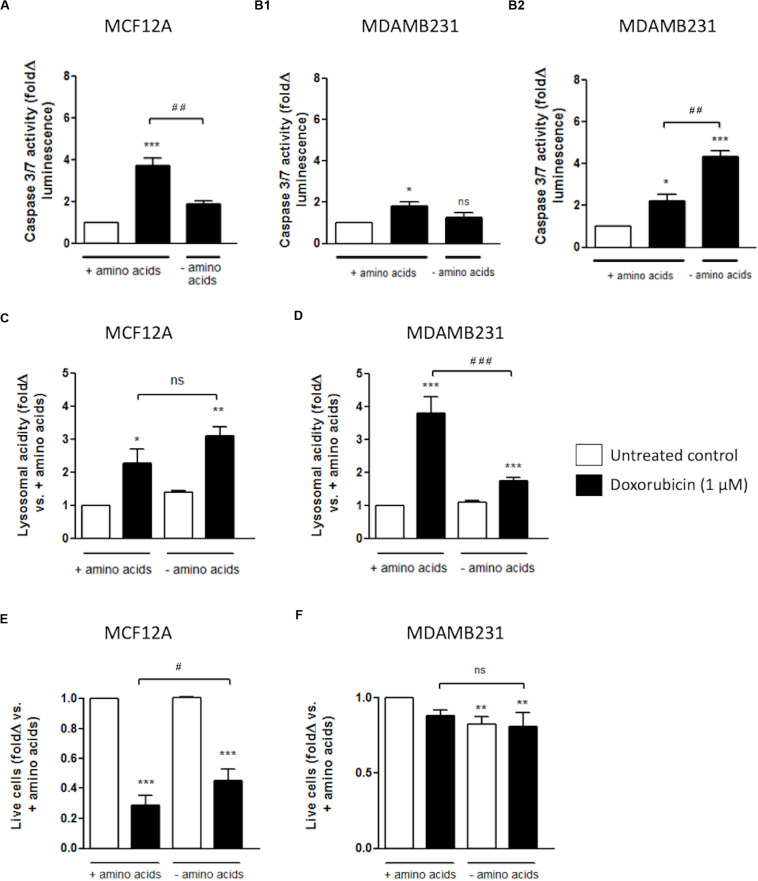
The effect of amino acid starvation in MCF12A and MDAMB231 cells following Dox treatment. Caspase 3/7 activity in amino acid-starved MCF12A after 24 h **(A)** and MDAMB231 cells after 12 **(B1)** and 24 h **(B2)** following Dox treatment. Lysosomal acidity in amino acid-starved MCF12A **(C)** and MDAMB231 **(D)** cells following Dox treatment. Trypan blue positive cell staining in amino acid-starved MCF12A **(E)** and MDAMB231 **(F)** cells following Dox treatment. Results represent the fold change in cultures incubated with Dox vs. untreated cells. Cells were incubated in Dox and/or in amino acid deprived medium for 24 h where applicable. Each value represents the mean ± SEM of at least three independent determinations. *p < 0.05; **p < 0.01; ***p < 0.001; ^#^*p* < 0.05; ^##^*p* < 0.01; ^###^*p* < 0.001; ns, no significance.

Amino acid deprivation significantly decreased caspase 3/7 activity in MCF12A cells when treated with Dox for 24 h (Figure 5A). This protection from increased caspase activation corresponded with increased and sustained lysosomal acidity, as measured with flow cytometry using cells stained with Lysotracker (Figure 5C). This is in direct contrast to the results obtained with MDAMB231 cells where incubation in culture medium deprived of amino acids, during treatment with Dox, resulted in significantly increased caspase 3/7 activity at 24 h (Figure 5B2), but not 12 h (Figure 5B1) after intervention. Amino acid deprivation during a 24 h treatment with Dox also resulted in significantly diminished lysosomal acidity levels, close to baseline, in MDABM231 cells (Figure 5D).

The trypan blue vital stain can traverse only cell membranes with compromised integrity, and therefore functions as a marker of necrotic and late stage apoptotic cell death and can be used as an indicator of actual cellular impairment following an intervention. MCF12A cells are extremely susceptible to Dox cytotoxicity with approximately 80% of these cells becoming trypan blue positive after a 24 h incubation with Dox (Figure 5E). Treatment with Dox in culture medium completely depleted of amino acids resulted in significantly less MCF12A cells becoming trypan blue positive.

MDAMB231 cells appeared to be comparatively resistant to Dox-induced membrane impairment, and amino acid starvation did not change the percentage of trypan blue positive MDAMB231 cells following treatment with Dox (Figure 5F).

### Amino Acid Deprivation Increased Autophagy Induction and Autophagy Flux in MCF12A Cells

**FIGURE 6:**
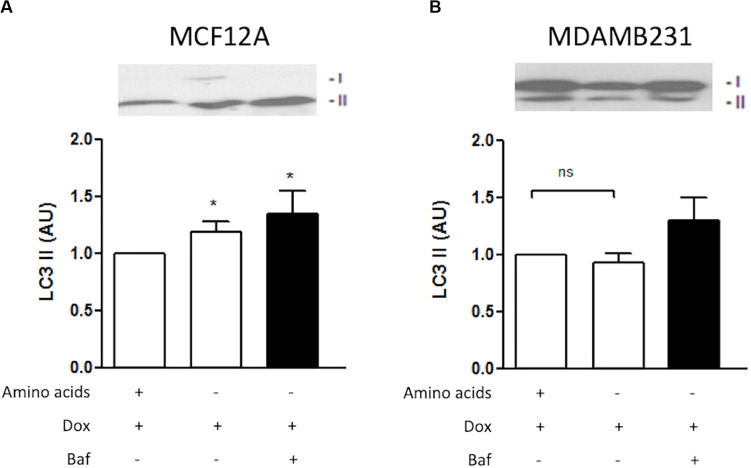
LC3 II protein levels during amino acid starvation in MCF12A and MDAMB231 cells. Representative western blots and Bar graphs showing densitometric representations of LC3 II protein levels in MCF12A **(A)** and MDAMB231 **(B)** cells. Each value represents the mean ± SEM of at least three independent determinations. AU, arbitrary units; *p < 0.05 vs. amino acid control; ns, no significance.

LC3-II decorates inner and outer membranes of autophagosomes and is the only reliable marker to analyze autophagy flux (Martinez-Lopez et al., 2016). Dox treatment during amino acid deprivation resulted in increased induction of LC3 II in MCF12A cells (Figure 6A). Furthermore, LC3 II protein levels were shown to accumulate if Baf was administered 6 h prior to analysis. Although a slight change was noticed in MDAMB231 cells when Baf was administered, this change was found to be non-significant (Figure 6B).

### Amino Acid Deprivation or Autophagy Inhibition With ATG5 siRNA During Dox Treatment Resulted in Increased Levels of Dox in the Nuclear Regions of MDAMB231 Cells

**FIGURE 7:**
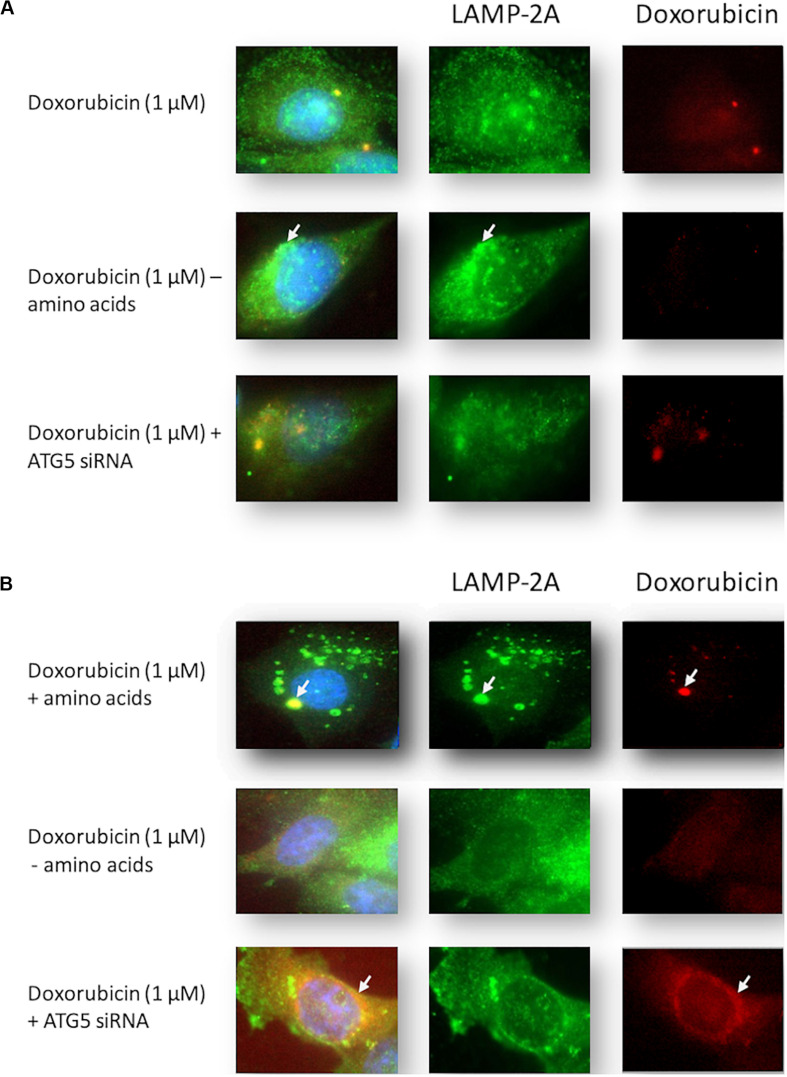
Intracellular localization of LAMP-2A and Dox in Dox-treated MCF12A and MDAMB231 cells following amino acid starvation and autophagy inhibition. Representative images of LAMP-2A (green) straining and Dox (red) in MCF12A **(A)** and MDAMB231 **(B)** cells treated with Dox in culture medium without amino acids or with ATG5 siRNA. Arrows highlight differences in localized signals of LAMP-2A and Dox. Comparative control groups (cells treated with Dox in the presence of amino acids) are shared between Figure 4 and Figure 7 since treatment groups from both figures were performed together in one experiment. Images were obtained using a 40× objective.

Amino acid deprivation from culture medium during treatment of MCF12A cells with Dox caused an apparent increase in pooled punctate LAMP-2A in the perinuclear area of these cells (illustrated by the arrow) (Figure 7A). Furthermore, amino acid deprivation during treatment resulted in a prominent decrease in observable Dox (red fluorescence). Inhibition of autophagy with ATG5 siRNA resulted in a dispersed pattern of LAMP-2A staining, but Dox was still readily visible in these cells.

MDAMB231 cells treated with Dox displayed intense localized regions of LAMP-2A and intracellular red fluorescence associated with each other (illustrated by the arrow) (Figure 7B). Amino acid deprivation during treatment resulted in a more dispersed staining pattern for the LAMP-2A marker. Furthermore, these cells displayed increased levels of nuclear Dox (red fluorescence). Inhibition of autophagy with ATG5 siRNA resulted in a prominent increase in intracellular Dox, especially at the perinuclear zone (illustrated by the arrow).

### Amino Acid Deprivation During Dox Treatment Exacerbated g2/m Cell Cycle Arrest Associated With Dox Toxicity in MDAMB231 Cells

**FIGURE 8:**
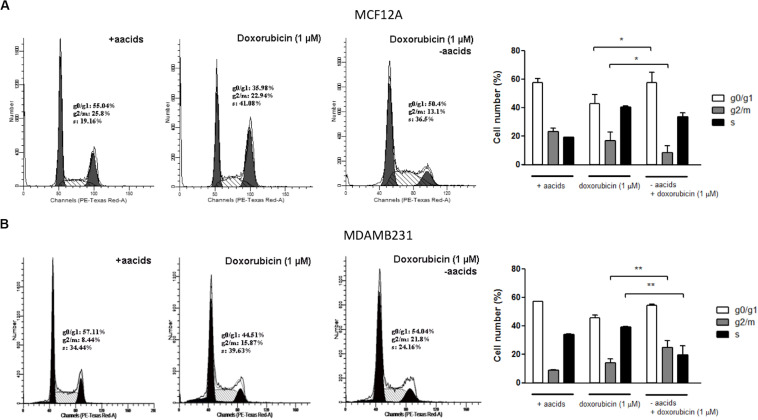
The effect of amino acid starvation and Dox treatment on the cells cycle of MCF12A and MDAMB231 cells. Representative DNA histograms and quantified bar graphs of cell cycle progression in MCF12A **(A)** and MDAMB231 **(B)** cells. Cell cycle progression was assessed using flow cytometry. Each value represents the mean ± SEM of at least three independent determinations. aacids, amino acids; *p < 0.05; **p < 0.01.

Dox treatment of MCF12A cells resulted in significant changes to the cell cycle profile. Deprivation of amino acids from culture medium during treatment with Dox resulted in the percentage of cells in the g0/g1 phase being similar to those in untreated controls (Figure 8A). However, the percentage of cells in g2/m phase decreased further if amino acids were absent.

Dox treatment is typically associated with an increased g2/m arrest in MDAMB231 cells (Lambert et al., 2008), although only a non-significant increase was observed in this specific model (Figure 8B). Treatment of MDAMB231 cells with Dox in culture medium deprived of amino acids resulted in a further significant increase in the percentage of cells in the g2/m phase of the cell cycle.

### Amino Acid Deprivation Does Not Alter Lysosomal Acidity or Apoptosis Levels During Dox Treatment in Beclin 1 Haploinsufficient MCF7 Cells

**FIGURE 9:**
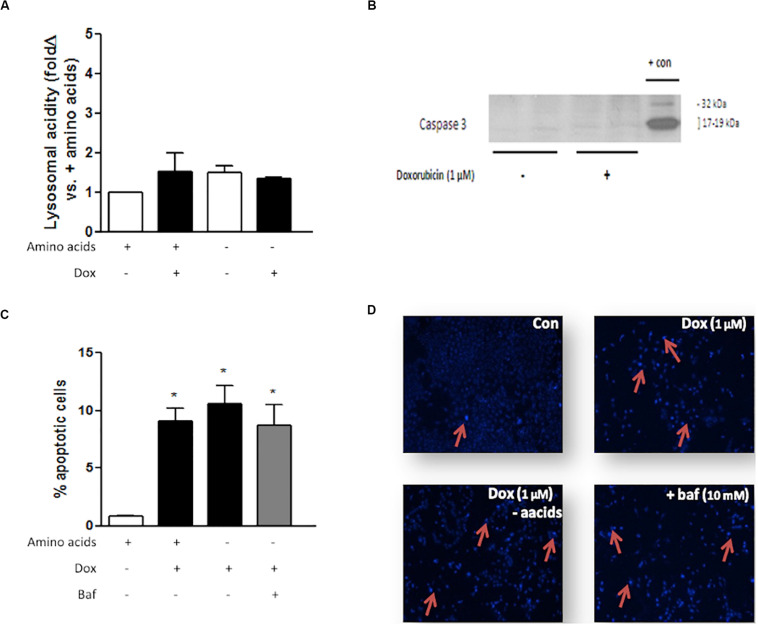
The effect of amino acid starvation, autophagy inhibition and Dox treatment in beclin 1-haploinsufficient MCF7 cells. **(A)** Lysosomal acidity in amino acid-starved MCF7 cells following Dox treatment. **(B)** Representative western blot showing lack of caspase 3 expression in MCF7 cells. **(C)** Bar graph indicating the percentage of nuclei that represents morphological changes characteristic of apoptosis in MCF7 cells following Dox treatment and Baf-mediated autophagy inhibition and **(D)** representative images of MCF7 cells stained with Hoechst. Red arrows demonstrate apoptotic nuclear features. Images were obtained using a 10× objective. Each value represents the mean ± SEM of at least three independent determinations. + con, positive control; con, control; aacids, amino acids; *p < 0.05 vs. amino acid control.

MCF7 cells are autophagy incompetent due to a haploinsufficiency in the gene coding for the autophagy protein beclin 1 (Liang et al., 1999). Dox treatment, either with or without amino acids, did not significantly alter lysosomal acidity in these cells (Figure 9A). MCF7 cells do not express caspase 3 (Liang et al., 1999), which was confirmed by western blotting analysis (Figure 9B). Dox treatment of MCF7 cells resulted in increased apoptosis (Figures 9C,D). However, Dox treatment of these cells in culture medium deprived of amino acids, either with or without Baf, does not significantly alter apoptosis levels.

### 24 h Protein Starvation During High Dose Doxorubicin (10 mg/kg) Treatment Resulted in Increased Survival of E0771 Tumor-Bearing GFP-LC3 Mice

**FIGURE 10:**
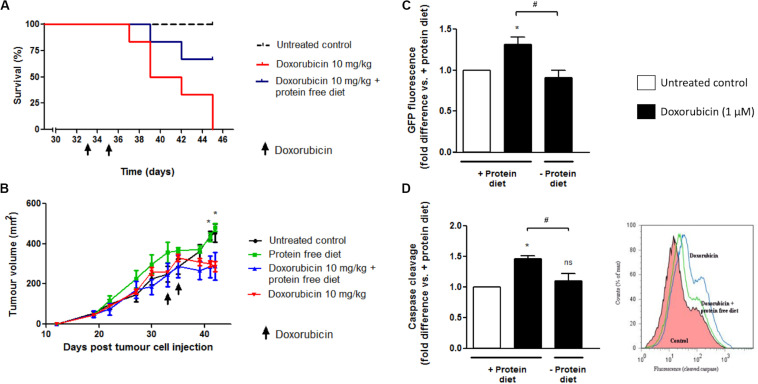
The effect of 24 h protein starvation during high dose doxorubicin (10 mg/kg) treatment on survival and tumor growth of E0771 tumor-bearing mice. Mice received two i.p. injections of doxorubicin (10 mg/kg) over a period of 3 days and were placed on either a isocaloric diet or a diet free of protein for a total 24 h immediately following each doxorubicin administration. **(A)** Survival of tumor-bearing mice following doxorubicin treatment with or without protein starvation, *N* = 6. **(B)** Changes in tumor volumes of tumor-bearing mice following doxorubicin treatment with or without protein starvation, *N* = 6. Values are expressed as mean ± SEM. *p < 0.05 untreated control vs. doxorubicin and vs. doxorubicin + protein free diet. **(C)** Intratumour autophagy flux in GFP-LC3 mice following doxorubicin treatment with or without protein starvation. A decrease in GFP signal indicates increased autophagy flux. *N* = 3. Values are expressed as mean ± SEM. *p < 0.05 vs. untreated + protein diet. ^#^*p* < 0.05. **(D)** Intratumour caspase cleavage in GFP-LC3 mice following doxorubicin treatment with or without protein starvation. FLIVO^TM^ caspase dye was used to assess caspase cleavage, with increased fluorescence indicating cleavage. *N* = 3. Values are expressed as mean ± SEM. *p < 0.05 vs. + protein diet. *p < 0.05; ^#^*p* < 0.05; ns, no significance.

Mice with similar tumors and administered doxorubicin at a high dose (10 mg/kg) on 2 days, either side of a day without treatment had all died by day 12 after the initial treatment (Figure 10A). The dose of doxorubicin was selected for acute exposure on the basis of previous work that verified an inevitably toxic but not rapidly fatal dose inducing myocardial damage, which is a common side-effect of doxorubicin therapy (Sishi et al., 2013).

However, if tumor-bearing mice were placed on a diet free of protein, immediately after i.p. injection with a high dose of doxorubicin (10 mg/kg), then the survival of these mice was prolonged compared to those fed a standard diet. Furthermore, protein starved doxorubicin treated mice showed less signs of reduced mobility and ruffled hair compared to mice treated with doxorubicin but fed isocaloric protein complete diets (data not shown).

### 24 h Protein Starvation During High Dose Doxorubicin (10 mg/kg) Treatment Does Not Influence Changes in Tumor Volumes Attributed to Doxorubicin

Growth rates of E0771 tumors in GFP-LC3 mice were very similar in size between groups, prior to the initial interventions (Figure 10B). Administration of two high dose doxorubicin (10 mg/kg) treatments over 3 days in GFP-LC3 mice bearing large E0771 tumors (>230 mm^2^) resulted in significant reductions in tumor size by day eight after the first intervention (Figure 10B). If tumor-bearing mice were placed on diets free of protein during doxorubicin treatment (10 mg/kg), reductions in tumor size were shown to be similar to those treated with doxorubicin but fed protein complete diets (Figure 10B).

### 24 h Protein Starvation During High Dose Doxorubicin (10 mg/kg) Treatment Results in Significantly Increased Intratumour Autophagy Flux in E0771 Induced Tumors in GFP-LC3 Mice

Flow cytometry and FACS can be used to monitor autophagy in cells containing GFP-LC3 by exploiting the fact that GFP is sensitive to acidic environments, such as that of lysosomes (Martinet et al., 2006; Yang et al., 2007), and GFP fluorescence disappears immediately on it entering the reduced pH environment of a lysosome. A reduction in GFP signal therefore reflects delivery of the GFP-LC3 complex into lysosomes and can be related to the degree of autophagy flux (Shvets et al., 2008). Administration of two high dose doxorubicin (10 mg/kg) treatments over 3 days in GFP-LC3 mice bearing large E0771 induced tumors (>230 mm^2^) resulted in a significant increase in GFP signal. This is inferred to indicate an increased autophagy induction and translation of the GFP-LC3 complex (Figure 10C). However, if mice were placed on a protein free diet for 24 h at the start of their night cycle, beginning immediately after each doxorubicin (10 mg/kg) injection, then a significant decrease in GFP signal indicates a significant increase in autophagy flux compared to mice treated but fed on protein complete diets. As only non-cancer cells contain the GFP-LC3, significantly increased intratumour autophagy flux implies that there is an increased autophagy flux in non-cancer stromal cells within the E0771 induced tumors of treated GFP-LC3 mice fed protein free diets compared to those fed protein complete diets.

### 24 h Protein Starvation During High Dose Doxorubicin (10 mg/kg) Treatment Results in Significantly Lower Caspase Activity Within Tumors

Administration of two high dose doxorubicin (10 mg/kg) treatments over 3 days in GFP-LC3 mice bearing large E0771 induced tumors (>230 mm^2^) resulted in a significant increase in caspase activity within whole excised tumors, using a FLIVO^TM^ (FLuorescence in vIVO) *in vivo* apoptosis tracer (Immunochemistry Technologies LLC, MN, United States) and FACS flow cytometry (Figure 10C). However, if mice were placed on a protein free diet for 24 h at the start of their night cycle, beginning immediately after each doxorubicin injection, then caspase activity was observed to be significantly lower in excised tumors than in those mice treated but fed on protein complete diets.

## Discussion

Many cancers are known to respond to certain chemotherapeutics or to radiation therapy by increasing autophagic activity. Although the consequences of autophagic activation in these circumstances are still debatable, it appears autophagy acts in its predominant role as survival mediator in many of these cases (Yang and Chen, 2011). Anthracyclins such as Dox have also been shown to increase autophagy levels in cancer cells in some instances. At lower doses, Dox elicits an autophagic response in breast cancer cells (Akar et al., 2008), as well as in sarcoma cell lines (Martinez-Lopez et al., 2016). Additionally, increased levels of ATG2A mRNA have been observed following Dox treatment (Levy and Thorburn, 2011), suggesting increased autophagy activation at the transcriptional level.

It was also reported that 2-deoxy-glucose preserved ATP content and contributed to the cytoprotection of rat cardiomyocytes treated with 1 μM Dox (Chen et al., 2011). This glucose analogue increased markers of autophagy which was posited as a potential reason for protection from cytotoxicity. Conversely, activation of autophagy in cultured cardiomyocytes following 1 μM Dox treatment mediated its cardiotoxic effect (Kobayashi et al., 2010). Here, it was shown that autophagy inhibition resulted in decreased cell death and it was postulated that autophagy directly contributed to Dox-induced toxicity. These exemplify the conflicting nature of studies and reported findings relating autophagy to drug toxicity and illustrate the need for further research into this important topic.

Autophagy activity is increased in response to stress, where it functions as an important mechanism whereby damaged organelles and aggregated proteins are degraded in lysosomes. However, there is still no clear mechanism whereby induction of autophagy could lead to tolerance against chemotherapy agents. Lysosomal fusion and throughput of autophagy depends on the low pH of lysosomal compartments (Klionsky et al., 2008). Increasing the lysosomal pH with pharmacological agents is an effective method of autophagy inhibition, as disruption of the fusion event between autophagosomes and lysosomes prevents progression of autophagy. Some drug resistant cell lines are able to tolerate alkaline chemotherapy drugs such as the anthracyclines by sequestering and deactivating these agents in the acidic compartments of lysosomes (Hurwitz et al., 1997). Exposure of these compounds to environments of low pH renders them inactive and unable to access sites of chemotoxicity such as the nucleus.

Data from the current study clearly indicated that the non-tumourigenic cell line MCF12A is more susceptible to programmed cell death during treatment with a moderate dose of Dox (1 μM) than the metastatic breast cancer cell line MDAMB231. MCF12A cells had a significantly increased appearance of morphological changes characteristic of apoptosis (Figure 1A), as well as significantly enhanced caspase 3/7 activity (Figure 3A). On the other hand, MDAMB231 cells showed few signs of late stage apoptosis and a more modest increase in activation of caspase 3/7 (Figures 1B, 3D). Interestingly, depletion of amino acids from the culture medium of MCF12A cells during Dox treatment resulted in a significant protection from loss of membrane integrity (a sign of late stage cell damage), as assessed by the trypan blue assay (Figure 5E). Previous experimental data revealed that amino acid starvation resulted in an enhanced autophagy response (unpublished from this group). It is suggested here that this increase in autophagy could be responsible, at least in part, for the tolerance to Dox treatment seen here. On the other hand, MDAMB231 cells did not experience any protection if starved of amino acids during treatment (Figure 5F).

Both MCF12A and MDAMB231 cells responded to the presence of Dox (1 μM) by increasing LC3 II protein levels (Figure 2), indicating an autophagic response during treatment. A slight increase in beclin 1 protein expression was also observed, however, these changes were not statistically significant. While beclin 1 plays an important role in the regulation of autophagy, it is also cleaved by caspase 3 during apoptosis (Wirawan et al., 2010). Since apoptosis was induced under these conditions, it may have resulted in varying levels of detectable beclin 1 protein expression and thus the lack of a significant effect. Inhibition of autophagy with ATG5 siRNA lead to a significant increase in caspase 3/7 activity only in MDAMB231 cells (Figure 3E), suggesting that autophagy has a protective role during chemotherapy of these cells. Importantly, the addition of Baf, 6 h prior to analysis, greatly increased caspase 3/7 activity during Dox treatment in both cell lines (Figures 3C,F), but had a particularly pronounced influence on the resistant cancer cell line. The differential effects displayed between ATG5 siRNA and Baf treatment in these cell lines might be due to the fact that Bafilomycin represents a mechanism of late-stage autophagy inhibition, while ATG5 knockdown facilitates autophagy inhibition at an earlier stage. Agents such as Baf, a specific inhibitor of vacuolar H^+^ATPase (V-ATPase) (Klionsky et al., 2008), are known to rapidly and reversibly inhibit fusion between autophagosomes and lysosomes, if administered for short time periods (Chen et al., 2011), through the mechanism of inhibiting lysosomal acidification (Hurwitz et al., 1997). Furthermore, many drug resistant cancer cells are thought to increase sequestration and deactivation of chemotherapy drugs within lysosomes (Kobayashi et al., 2010). The proposed mechanism is through increased uptake of these compounds into endosomes, which eventually fuse with lysosomes to deliver these drugs into the acidic internal environments where they become deactivated (Wirawan et al., 2010). Baf and other agents that raise the pH of lysosomes can increase cytotoxicity by preventing fusion and inactivation of drugs in this way. Therefore, the increased caspase activity observed in the current study, following Baf administration, suggested that Baf prevented the sequestration and deactivation of Dox, therefore allowing Dox to exert its cytotoxic effects on the cell. As autophagy inhibition also resulted in increased caspase activity here, it is possible that mass engulfment of cytoplasmic material (which includes Dox) could facilitate delivery of this drug into lysosomal compartments in this cell line and thereby have a protective influence. As MDAMB231 cells have high basal autophagy levels (unpublished data), internalized Dox could be rapidly delivered to lysosomes and deactivated, conferring a partial resistance.

These assertions are further strengthened by qualitative evidence gained from fluorescence microscopy of MDAMB231 cells receiving Dox treatment. Intracellular localization of Dox can be tracked by exploiting the autofluorescence of Dox, a technique that has been utilized successfully in MDAMB231 and other cell lines in the past (Yoshimori et al., 1991). Treatment of MDAMB231 cells resulted in the clear accumulation of Dox at perinuclear regions (Figure 4B). Identification of the localization of lysosomes at the same time, by fluorescently tagging the lysosomal associated membrane protein LAMP-2A, demonstrates lysosomal accumulation in regions associated with Dox accumulation in these cells. This implies that Dox is located in association with lysosomes during these conditions. MCF12A cells show little accumulation of Dox after a 24 h treatment and have no discernible accumulation of LAMP-2A signal (Figure 4A). Furthermore, the addition of Baf 6 h prior to imaging, led to a vast and pronounced accumulation of Dox within the cytoplasmic compartment of MDAMB231 cells. Interestingly, accumulation of the LAMP-2A signal is mostly dissipated and little association between LAMP-2A and Dox was evident after the addition of Baf. Therefore, it appears as though Dox is not associated with lysosomes when administered in the presence of the vacuolar H^+^ATPase inhibitor Baf, but rather elsewhere within the cell. As Baf inhibits the fusion of autophagosomes and endosomes with lysosomes (Hurwitz et al., 1997; Chen et al., 2011), it is possible that the observed accumulations of Dox is due to increased cytoplasmic accumulation of autophagosomes and endosomes containing Dox, but which are unable to fuse with lysosomes. Further investigation of this phenomenon is required.

Autophagic flux is clearly increased in MDAMB231 cells in response to Dox treatment (Figure 6), and lysosomal acidity greatly increased as a result of this treatment in both MCF12A cells (Figure 5C) and in MDAMB231 cells (Figure 5D). Interestingly, when these cell lines were treated with Dox in culture medium deprived of amino acids, lysosomal acidity significantly decreased in MDAMB231 cells, but remained elevated in MCF12A cells. Notably, alterations in lysosomal acidity were associated with corresponding changes in caspase 3/7 activity in both cell lines (Figures 5A,D). MDAMB231 cells experienced significantly greater levels of caspase 3/7 activity during conditions of depressed lysosomal acidity, while MCF12A cells were granted a relative protection from the cytotoxic impact of drug treatment. Strikingly, if MDAMB231 cells were analyzed after 12 h of combined Dox/amino acid starvation treatment, these increases in apoptosis activation were not evident. Importantly, at this time point of amino acid starvation lysosomal acidity was confirmed to be elevated compared to baseline (unpublished data), further supporting the premise of lysosomal acidity deprivation facilitated increases in caspase activity in this model.

If decreased lysosomal acidity levels during amino acid starvation prevent fusion of autophagosomes with lysosomes then the probability of drug access to sites of cytotoxic action would be increased. This would result in the increased apoptosis activation demonstrated here. Dox treatment is associated with genotoxic stress and prolonged g2/m cell cycle arrest (Yamamoto et al., 1998), due to intercalation of this agent with DNA and activation of the g2/m DNA damage checkpoint. Therefore, decreased abundance of Dox within lysosomes during amino acid deprivation would result in an exacerbated accumulation of cells in the g2/m phase of the cell cycle. Our experimental data supports this with an approximately 6% increase in the percentage of MDAMB231 cells in the g2/m phase of the cell cycle if Dox treated cells are simultaneously starved of amino acids (Figure 8B). Additionally, the relative protection of MCF12A cells can be attributed to increased autophagy levels and decreased access of Dox to the nucleus, as amino acid starvation during drug treatment resulted in a cell cycle profile similar to that of untreated cells (Figure 8A). Also, qualitative experimental evidence shows that Dox treatment during amino acid starvation causes decreased Dox and LAMP-2A accumulation and an increased Dox signal in the nuclear regions of MDAMB231 cells (Figure 7B), whereas the opposite is observed in MCF12A cells during similar treatment (Figure 7A).

Data suggested that alterations in lysosomal acidity are linked to increased apoptosis induction or protection in MDAMB231 cells and MCF12A cells, respectively. However, the role for autophagy should not be overlooked. Inhibition of autophagy with ATG5 siRNA resulted in a prominent increase in levels of Dox in the nucleus and at the perinuclear zone in MDAMB231 cells (Figure 7B), while MCF12A cells exposed to ATG5 siRNA prior to treatment showed signs of increased cytoplasmic Dox accumulation (Figure 7A). Interestingly, a cell line with decreased autophagy (MCF7), due to haploinsufficiency in the gene coding for beclin 1, displayed no alterations in lysosomal acidity during Dox treatment, in the presence or absence of amino acids (Figure 9A). While this cell line does not possess active caspase 3 (Figure 9B), it did show signs of significantly increased apoptosis during Dox treatment. These levels of apoptosis did not increase if treatment occurred in the absence of amino acids or the presence of Baf (Figures 9C,D). Together, these results indicate that active autophagic machinery is required for the accumulation of Dox within lysosomes and demonstrated an important role for autophagy in these processes. As interest in modulation of autophagy during cancer treatment increases and novel strategies such as fasting therapy during high-dose cancer treatment are explored, it is imperative that studies be undertaken to understand the underlying mechanisms driving these beneficial effects before use begins in a clinical setting.

Therefore, in this part of the study we aimed to determine the effect of short-term protein starvation in mice receiving a high cumulative dose of doxorubicin treatment. Using a mammary tumor model, it was shown that high-dose doxorubicin treatment (10 mg/kg for a cumulative dose of 20 mg/kg over 3 days) resulted in low survival rates of rodents possessing large, aggressive tumors (Figure 10A). Remarkably, survival was significantly improved if mice were placed on protein free diets immediately after drug administration (Figure 10A). Although evidently extremely toxic to the tumor-bearing mice in this model, the dose of doxorubicin used here was sufficient to significantly reduce tumor size after only 8 days. However, these reductions in tumor sizes were not diminished in those mice starved of proteins during treatment (Figure 10B).

Since our *in vitro* experimental data has shown that while a non-cancer cell line experienced protection if starved of amino acids during doxorubicin treatment, a cancer cell line with high basal autophagy activity had increased cell death. We have therefore utilized our GFP-LC3 tumor-bearing mouse model to assess the impact of protein starvation on autophagy flux in the host derived stromal subpopulation within the mammary tumors of mice treated with doxorubicin. We have shown an increase in intratumour autophagic flux in the non-cancer cell population of these tumors (Figure 10C). Interestingly, this increase in autophagy flux in the tumor stromal subpopulation correlated with a decrease in intratumour apoptosis (Figure 10D).

Cancers are extremely heterogenous by nature and much of their volume can be comprised of non-cancer cells that are vital for their continued growth and development (Lee and Tannock, 2006). As solid tumors rely on non-cancer cells for survival, any protection of these cells from cytotoxicity during short term starvation could indirectly result in prolonged tumor cell survival during chemotherapy. Also, the benefits of fasting in cellular protection from cytotoxicity may rely partly on altered circulating hormone levels (Li et al., 2010), which necessitates further investigation using *in vivo* models. In fact, reduced circulating IGF-I levels have been implicated in the differential protection of normal cells and cancer cells in response to fasting and improved chemotherapeutic index during doxorubicin treatment (Koutsilieris et al., 1999).

**FIGURE 11:**
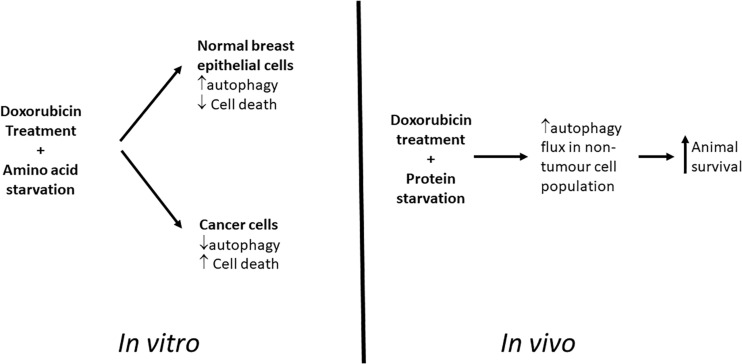
Graphical Summary. Proposed model for differential toxicity in normal breast epithelial cells and breast cancer cells.

Our cell culture based studies demonstrated a promising differential protection of non-cancer cells and increased signs of apoptosis in cancer cells during chemotherapy when these cell lines were starved of amino acids (Figure 11). However, translation of this model in vivo has shown that although protein deprivation appears to increase survival rates without impacting on reductions in tumor volumes during high dose doxorubicin treatment, a potentially increased protection may be occurring in cells within these tumors. The model established here, and the related findings, have presented a novel and unique platform for further research into this remarkable phenomenon, and particularly the role of autophagy therein.

This data represents a caveat to researchers aiming to utilize fasting or starvation diets in combination with conventional chemotherapy, and illustrates that additional mechanistic data is required in order to better understand the indirect consequences of such treatment strategies. Together with the *in vitro* experimental data, these promising findings imply a role for autophagy in the differential protection during doxorubicin treatment.

## Conclusion

The results of this study suggest that the inherent resistance to the alkaline chemotherapeutic agent Dox exhibit by the MDAMB231 cells can be attenuated by elevating the lysosomal pH in a pharmacological manner. Furthermore, short term amino acid starvation was shown to be a realistic avenue for adjuvant therapy since it strengthened the cytotoxic effect of Dox in the MDAMB231 cancer cells but not in the normal MCF12 cells. Non-cancerous cells were potentially protected from the anthracycline due to the ability to increase autophagic activity in response to short term amino acid starvation.

## Data Availability Statement

The raw data supporting the conclusions of this article will be made available by the authors, without undue reservation.

## Ethics Statement

The animal study was reviewed and approved by Animal Ethics Committee Stellenbosch University.

## Author Contributions

MT contributed to the conception and design of the study, and collected and analyzed data. TD drafted the initial manuscript. TN contributed to critical revision and intellectual input of the manuscript. BS participated in the animal study and contributed to critical revision. A-ME contributed to the conceptualization and design of the entire study, and supervised and contributed to critical revision and intellectual input of the manuscript. All authors read and approved the final manuscript.

## Conflict of Interest

The authors declare that the research was conducted in the absence of any commercial or financial relationships that could be construed as a potential conflict of interest.

## Supplementary Material

Supplementary Figure 1 The effects of ATG5 siRNA on MCF12 and MDAMB231 cells. Transfection of **(A)** MCF12A and **(B)** MDAMB231 cells with ATG5-targetting siRNA does not have any effect on caspase 3/7 activity under control conditions. Results represent the fold change in luminescence compared to untransfected cells and are proportionate to caspase activity. Each value represents the mean ± SEM of at least three independent determinations.
