# Supplementary figures and images for: Amino Acid Starvation Sensitizes Resistant Breast Cancer to Doxorubicin-Induced Cell Death
Source: Front Cell Dev Biol. 2020 Oct 15;8:565915. doi: 10.3389/fcell.2020.565915 (PMC7593593; doi:10.3389/fcell.2020.565915)

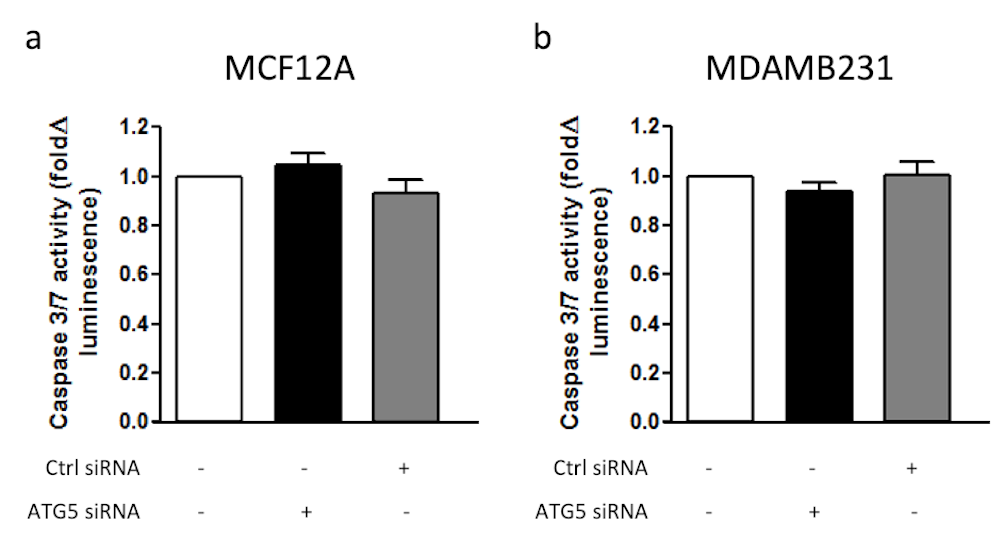

Supplement: Supplementary Figure 1 — The effects of ATG5 siRNA on MCF12 and MDAMB231 cells. Transfection of (A) MCF12A and (B) MDAMB231 cells with ATG5-targetting siRNA does not have any effect on caspase 3/7 activity under control conditions. Results represent the fold change in luminescence compared to untransfected cells and are proportionate to caspase activity. Each value represents the mean ± SEM of at least three independent determinations. [file Image_1.tif]
